# A Mass Spectrometry-Based Study Shows that Volatiles Emitted by *Arthrobacter agilis* UMCV2 Increase the Content of Brassinosteroids in *Medicago truncatula* in Response to Iron Deficiency Stress

**DOI:** 10.3390/molecules24163011

**Published:** 2019-08-20

**Authors:** Idolina Flores-Cortez, Robert Winkler, Arturo Ramírez-Ordorica, Ma. Isabel Cristina Elizarraraz-Anaya, María Teresa Carrillo-Rayas, Eduardo Valencia-Cantero, Lourdes Macías-Rodríguez

**Affiliations:** 1 Instituto de Investigaciones Químico Biológicas, Universidad Michoacana de San Nicolás de Hidalgo. Edifico B3, Ciudad Universitaria, Morelia 58030, Michoacán, Mexico; 2 Department of Biotechnology and Biochemistry, Cinvestav Unidad Irapuato, Irapuato, Km 9.6 Libramiento Norte Carr. Irapuato-León, Guanajuato 36824, Mexico

**Keywords:** legumes, microbial volatiles, Fe deficiency, DLI-ESI-MS

## Abstract

Iron is an essential plant micronutrient. It is a component of numerous proteins and participates in cell redox reactions; iron deficiency results in a reduction in nutritional quality and crop yields. Volatiles from the rhizobacterium *Arthrobacter agilis* UMCV2 induce iron acquisition mechanisms in plants. However, it is not known whether microbial volatiles modulate other metabolic plant stress responses to reduce the negative effect of iron deficiency. Mass spectrometry has great potential to analyze metabolite alterations in plants exposed to biotic and abiotic factors. Direct liquid introduction-electrospray-mass spectrometry was used to study the metabolite profile in *Medicago truncatula* due to iron deficiency, and in response to microbial volatiles. The putatively identified compounds belonged to different classes, including pigments, terpenes, flavonoids, and brassinosteroids, which have been associated with defense responses against abiotic stress. Notably, the levels of these compounds increased in the presence of the rhizobacterium. In particular, the analysis of brassinolide by gas chromatography in tandem with mass spectrometry showed that the phytohormone increased ten times in plants grown under iron-deficient growth conditions and exposed to microbial volatiles. In this mass spectrometry-based study, we provide new evidence on the role of *A. agilis* UMCV2 in the modulation of certain compounds involved in stress tolerance in *M. truncatula*.

## 1. Introduction

Mass spectrometry (MS) is gaining considerable popularity for profiling metabolites in complex biological samples. The increased applications have led to the improvement of MS technology in sample introduction, ionization source, mass analyzer, ion detection, and data acquisition and processing. Direct liquid introduction-electrospray ionization-mass spectrometry (DLI-ESI-MS, the acronym recommended by the Analytical Methods Committee [1]) is a rapid and high-throughput analytical tool that has been successfully applied in medicine and food and biological sciences [2,3,4,5]. DLI-ESI-MS does not require preliminary sample separation, and it can be applied to multiple biological matrices. The straightforward sample introduction allows for simultaneous fingerprinting of a vast number of metabolites from different samples within a single period. In addition, different studies support the repeatability of DLI-ESI-MS data, and the quantification of the intensity of the ion signals (*m*/*z*) with a larger variance in plants because of environment, physiological state, or the genotype can also be performed [5,6,7]. High analytical performance (sensitivity, selectivity) allows it to be used for untargeted metabolomics screening approaches for different plant extracts; thus it offers an excellent cost-benefit ratio compared to other analytical platforms such as near-infrared reflectance spectroscopy (NIRS), ultra-performance liquid chromatography-mass spectrometry (UPLC-MS), gas chromatography with flame ionization detection (GC-FID), and GC-MS, which are slow and expensive to use for routine plant biochemistry studies [2]. Due to the various benefits reported for DLI-ESI-MS, we decided to conduct a study to determine its usefulness in microbial ecology research, as DLI-ESI-MS provides robust chemical information, is bioinformatically easy to handle, and could help us understand the chemical response of plants to abiotic or biotic factors. 

Iron (Fe) is an essential micronutrient for plant growth and crop productivity. Plants acquire Fe mainly from the rhizosphere; therefore, the mechanisms that regulate Fe acquisition and homeostasis in the plant are of interest. The role of specific metabolites such as nitric oxide, ferritin, phenolic compounds, and brassinosteroids (BRs) have been highlighted in Fe-deficient growth conditions, indicating that plants undergo significant metabolic changes during Fe-adaptive processes [8,9,10,11,12,13,14,15,16,17].

In an attempt to make agriculture a viable component of a healthy and pleasant ecosystem, the application of plant growth-promoting rhizobacteria (PGPR) to enhance Fe uptake and transport in plants is an excellent biotechnology strategy. PGPR are soil bacteria that colonize the rhizosphere of plants, stimulating plant growth and health through different mechanisms, such as phosphorus solubilization or nitrogen-fixation, and the production of phytohormones or siderophores to capture Fe from the environment in biologically useful forms [18]. In 2003, Ryu et al. reported that some PGPR can modulate the growth and development of plants without physical contact with them. This mechanism involves the production of volatile compounds such as, acetoin and 2,3-butanediol, which modulate the mechanisms of phytohormone signaling and therefore stimulate morphogenesis programs in plants [19]. Six-years later, Zhang et al. (2009) noted, that the same microbial volatiles can modulate Fe uptake in *Arabidopsis* via deficiency-inducible mechanisms [20]. 

The rhizobacterium *Arthrobacter agilis* UMCV2 used in this study, was isolated from the rhizosphere of maize (*Zea mays*) [21]. It emits a pool of volatiles that promotes the growth of leguminous and monocotyledonous plants with different levels of available Fe [22,23,24]. Notably, in *Medicago truncatula*, the UMCV2 strain increased the expression of genes involved in Fe uptake (*MtFRO1*, *MtFRO2*, *MtFRO3*, *MtFRO4*, and *MtFRO5*) under Fe-sufficient and -deficient conditions [25]. Nevertheless, those studies focused on elucidating the molecular mechanisms involved in the modulation of Fe acquisition responses. Thus, one question remaining is whether microbial volatiles modulate the production of other primary or secondary metabolites in plants to ameliorate Fe-deficiency stress.

Here, we used a DLI-ESI-MS method as an untargeted mass spectrometry strategy to study the metabolic profiles of *M. truncatula* seedlings grown under Fe-sufficient and -deficient conditions. We focused on the detection of significant differences among MS profiles to determine whether volatiles emitted by *A. agilis* UMCV2 alleviate plant stress and stimulate the accumulation of metabolites involved in abiotic stress tolerance; in addition, we used a complementary GC-MS method to confirm the identification of brassinolide, which is involved in Fe-adaptive processes in plants.

## 2. Results

### 2.1. DLI-ESI-MS in the Analysis of Fe Deficiency in Medicago truncatula Seedlings and Response to Bacterial Volatiles

**Figure 1:**
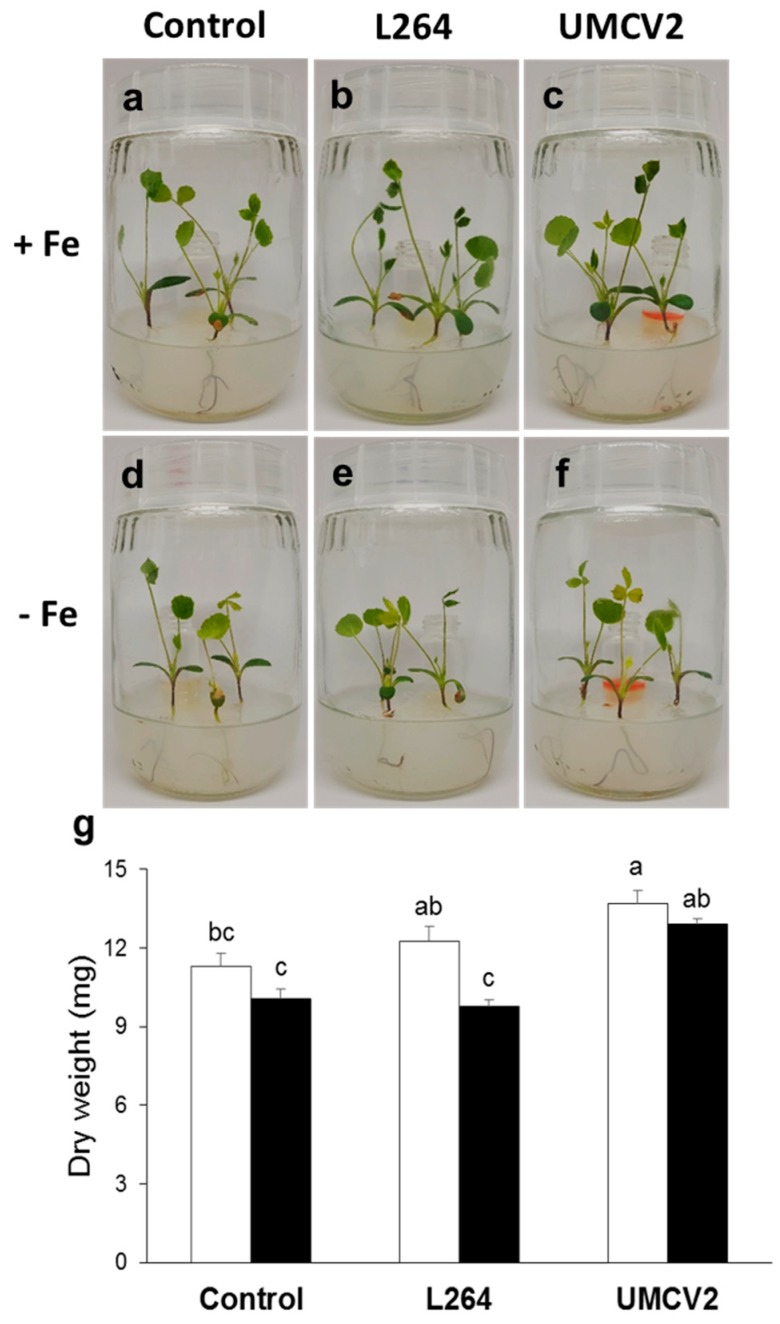
Interaction between *Medicago truncatula* and rhizobacteria through the emission of volatile compounds. A 4 mL glass vial with 2 mL nutritive agar medium was inserted in each system; in the control system, 20 μL water was added into the vial instead of the bacterial inoculum. The interaction lasted for 10 days. Uninoculated 12-day-old plants grown under conditions of iron (Fe) sufficiency (**a**) and deficiency (**d**). (**b**) Plants were inoculated with the commensal strain *Bacillus* sp. L264 grown under Fe-sufficient and -deficient conditions (**e**). Inoculated plants exposed to volatiles from *A. agilis* UMCV2 and under Fe-sufficient (**c**) and -deficient conditions (**f**). (**g**) Dry weights of control plants and plants during interactions with bacterial volatile compounds. Data shown are means ± standard error (n = 15). White and black bars indicate Fe-sufficient and -deficient growth conditions, respectively. Different letters indicate significant differences (*p* ≤ 0.05) among treatments determined with two-way ANOVA and Tukey’s test.

Under conditions of Fe deficiency, plants adjust their metabolism to maintain cellular Fe homeostasis. Some visual symptoms of Fe deficiency, such as leaf yellowing (Figure 1d–f) and decreased plant size (Figure 1g) were observed in our experiments in comparison to plants grown under Fe sufficiency (Figure 1a–c). Additionally, we studied plants exposed to volatiles from *A. agilis* UMCV2, a rhizobacterium that induces Fe acquisition in plants (Figure 1c,f), and plants exposed to volatiles from *Bacillus* sp. L264, a commensal rhizobacterium (Figure 1b,e). As we expected, volatiles from the UMCV2 strain, had a stimulatory effect on plant growth under Fe-sufficient and -deficient growth conditions (Figure 1g). 

**Figure 2:**
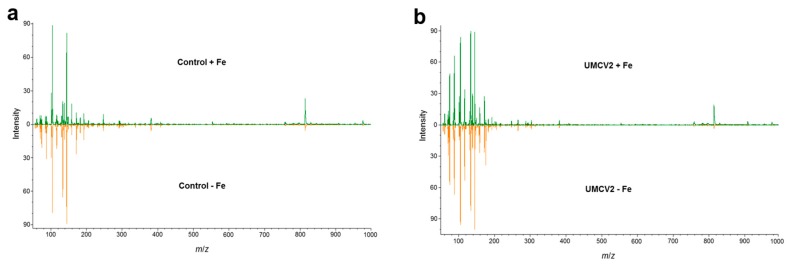
Non-targeted metabolomic profiling normalized from leaves of *Medicago truncatula* obtained by DLI-ESI-MS. (**a**) Control plants grown under Fe-sufficient (green) and -deficient conditions (orange). (**b**) Plants exposed to volatile compounds from *A. agilis* UMCV2 for 10 days and grown under iron-sufficient (green) and -deficient conditions (orange).

**Figure 3:**
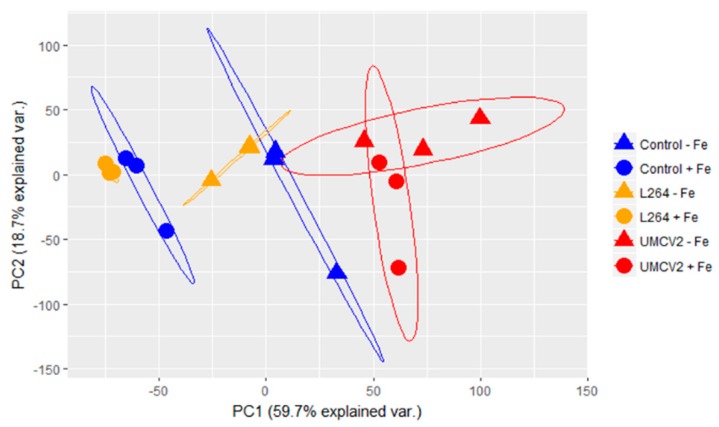
Principal component analysis (PCA) obtained from DLI-ESI mass spectra of *Medicago truncatula* leaves under Fe-sufficient and -deficient conditions and following exposure to microbial volatiles. Blue indicates control plants, orange represents plants exposed to volatiles emitted by L264 strain, and red shows the plants exposed to volatiles from *A. agilis* UMCV2. Circles (O) and triangles (Δ) indicate Fe sufficiency and deficiency, respectively. The ellipses represent 95% confidence intervals. Differences between groups were compared with a PERMANOVA test (*p* < 0.001).

These plants were further analyzed by DLI-ESI-MS. The quadrupole analyzer allowed the collection of MS data with satisfactory spectral quality. Typical mass spectra of the broad range of molecular weights of compounds that are produced when plants undergo Fe stress, and microbial volatiles exposure are shown in Figure 2a,b, respectively. In total, 737 ions were obtained, mainly within the range 55.90–1592.52 *m*/*z*. All metabolite signals were extracted from a database with the MALDIquant package in the RStudio interface. Following purification, alignment, and normalization, a principal component analysis (PCA) was performed (Figure 3). The PCA (highly significant results *p* < 0.001, by permutational multivariate analysis of variance, PERMANOVA) showed that control plants grown under conditions of Fe sufficiency and those exposed to volatiles from L264 had similar mass spectra, since treatments were grouped together. Similarly, plants grown under Fe-deficiency stress and those exposed to volatiles from L264 had the same metabolic fingerprinting, and both treatments presented an overlap, indicating that only the absence of Fe affected the metabolic profile of the plants. The ion profiles of plants grown under conditions of Fe sufficiency and deficiency, and following exposure to UMCV2 volatiles were similar, indicating that UMCV2 promotes metabolic changes in plants under both growing conditions.

### 2.2. Classification of Random Forest Model for Differentiating Plants Grown under Two Different Fe Conditions and Rhizobacterial Inoculation

**Figure 4:**
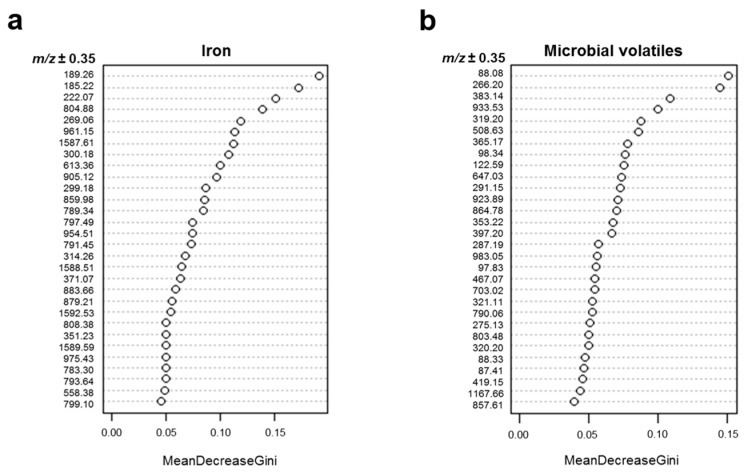
Ion importance ranking obtained by Random Forest model for differentiating between sample treatments with DLI-ESI mass spectra data. The thirty most important ions are shown for discriminating between Fe growth conditions (**a**) and the effect of microbial volatiles (**b**). Ntrees = 500, OOB error = 0% for Fe growth conditions and 43.75% for volatiles.

**Figure 5:**
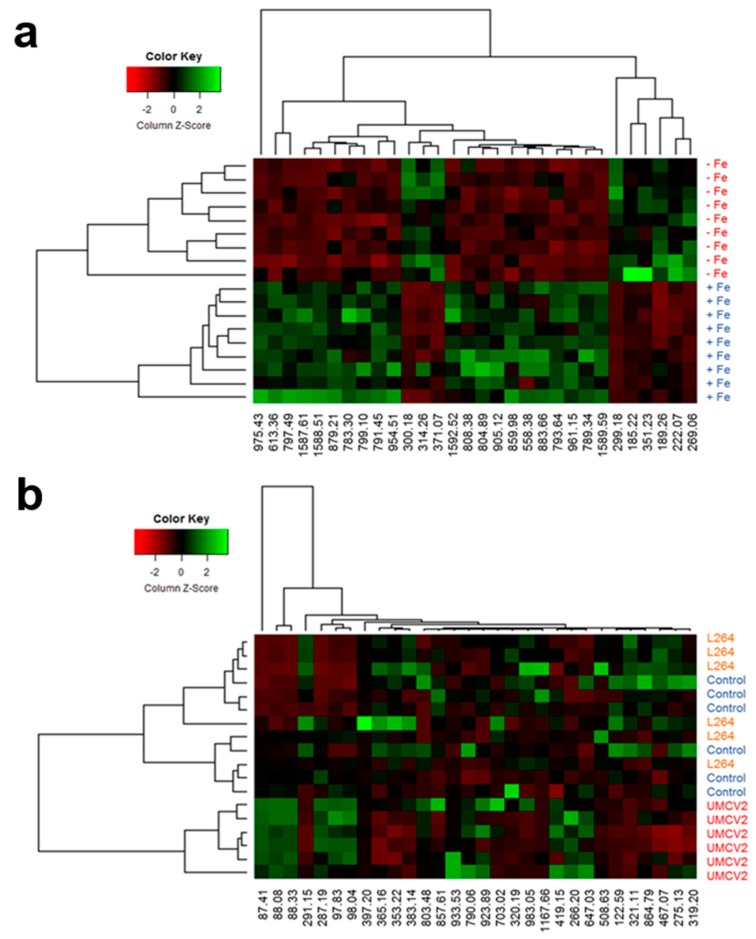
Metabolomic heatmap generated with the 30 most important ions detected by the Random Forest model for Fe availability (**a**) and bacterial volatiles (**b**). Heatmap combined with an analysis of cluster hierarchical using Euclidean distance between experimental units and Ward’s algorithm for classification by ion (along x-axis) and by treatment (along *y*-axis).

In order to reduce data complexity, the Random Forest (RF) algorithm was used to generate decision trees and extract the 30 most important ions, defining whether seedling samples were Fe-sufficient or -deficient (Figure 4a). Ions with the greatest mean decrease in Gini index were putatively identified using the PlantCyc database and SpiderMass software. The *m*/*z* from each ion was compared to the monoisotopic mass (Da) of metabolites previously reported for *M. truncatula*, which provided knowledge about participating metabolites in response to Fe-deficiency stress. Among the most important ions, we identified compounds involved in riboflavin metabolism at 185.22 *m*/*z* (1-deoxy-L-glycero-tetrulose 4-phosphate), 299.18 *m*/*z* [5-amino-6-(d-ribitylamino) uracil], and 808.38 *m*/*z* (flavin adenine dinucleotide, FAD); lipid metabolism at 797.49 *m*/*z* (1-18:3-2-18:3-monogalactosyldiacylglycerol), 859.98 *m*/*z* (butanoyl-CoA); and chlorophyll metabolism at 222.07 *m*/*z* (phosphonothreonine), 613.36 *m*/*z* (protochlorophyllide a) [11,26,27]. In addition, compounds that alleviate abiotic stresses in plants were also identified at 189.26 *m*/*z* (norspermine), 269.06 *m*/*z* ((+)-marmesin), 300.18 *m*/*z* ((*S*)-*N*-methylcoclaurine), 314.26 *m*/*z* (9,10-epoxy-18-hydroxystearate), 351.23 *m*/*z* (crocetin), and 371.07 *m*/*z* (chelerythrine) (Table 1). Of these ions, 185.22, 189.26, 222.07, 269.06, 299.18, and 351.23 *m*/*z* (Figure 5a) were detected at a higher intensity under Fe deficiency conditions. The remaining 17 ions selected by the RF algorithm for the Fe condition could not be identified. 

The RF model also helped to identify the 30 most important ions including those that differentiated uninoculated seedlings, and those inoculated with L264 or UMCV2 strains (Figure 4b). Twenty ions were identified (Table 2). Six of these, 87.41 *m*/*z* (3-pentanone), 88.08 *m*/*z* (pyruvate), 88.33 *m*/*z* (4-aminobutanal), 97.83 *m*/*z* (glycolate), 98.04 *m*/*z* (*N*-monomethylethanolamine), and 287.19 *m*/*z* (kaempferol) showed stronger detectable signal intensities under UMCV2 treatment (Figure 5b), suggesting that these ions are responsible for the discrimination between the sample groups, and revealing the associated chemical modulations made by the UMCV2 strain. Thus, DLI-ESI-MS displays great potential for determining whether volatiles emitted from other rhizobacteria are able to modulate the production of primary or secondary metabolites in plants. According to previously reported literature, the increased signals have different roles in alleviating Fe deficiency stress in plants [12,28,29,30,31,32,33]. Other identified compounds included those involved in plant primary metabolism, at 88.08 *m*/*z* (pyruvate), 97.83 *m*/*z* (glycolate), 790.06 *m*/*z* (coenzyme A) [34]; brassinosteroid metabolism, at 397.20 *m*/*z* (5-dehydroepisterol), 419.15 *m*/*z* (6-deoxocathasterone), and 467.07 *m*/*z* (6-alpha-hydroxycastasterone) [35,36,37,38]; compounds with antioxidant roles in plants, at 266.20 *m*/*z* (thiamine), 366.17 *m*/*z* (galactinol), and 933.53 *m*/*z* (notoginsenoside R1) [39,40,41], and some flavonoids with antioxidant capacity, which act as chemotactic signals for symbiotic nitrogen-fixing bacteria of legumes, at 275.13 *m*/*z* (fustin), 287.19 *m*/*z* (kaempferol), and 291.15 *m*/*z* (formononetin) [10,42,43,44,45] (Table 1). 

**Figure 6:**
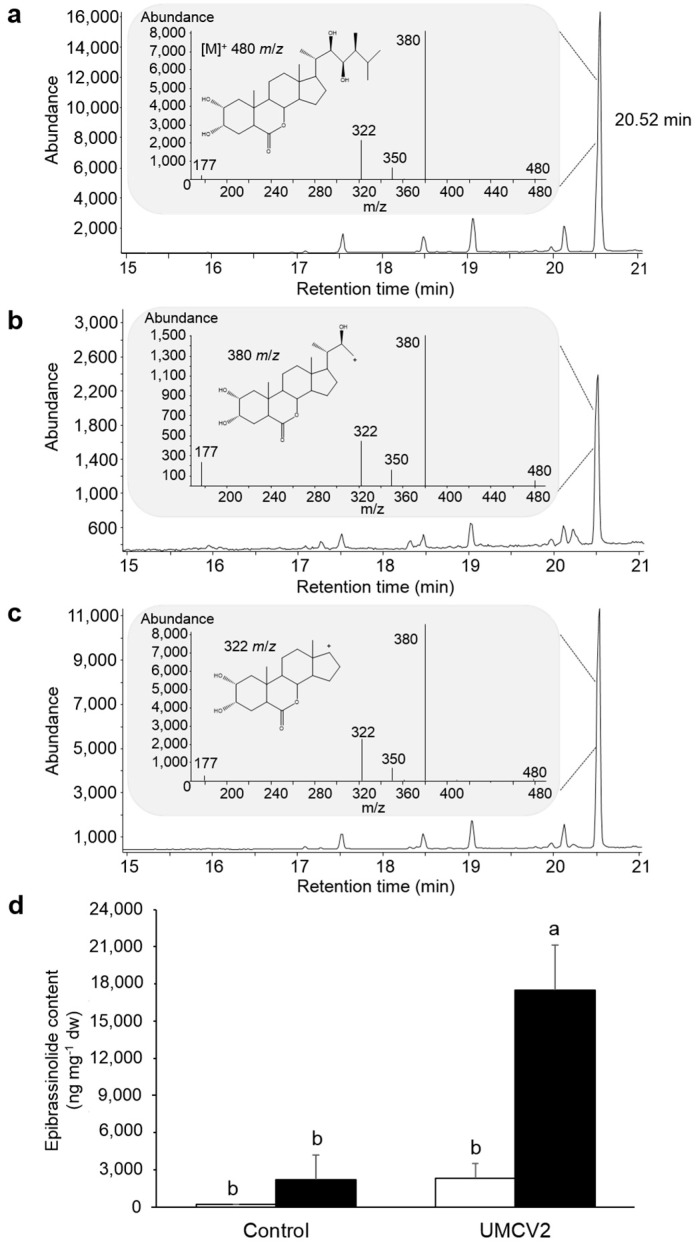
Identification of brassinolide in *Medicago truncatula* by GC-MS. (**a**) Total ion chromatogram of the epibrassinolide standard, indicating the retention time of the phytohormone and the electron impact mass spectrum during the SIM analysis. (**b**) Total ion chromatogram and mass spectrum obtained from the control plants grown under conditions of iron (Fe) deficiency. (**c**) Total ion chromatogram and mass spectrum obtained from plants grown under conditions of Fe deficiency and exposed to volatiles from *A. agilis* UMCV2. (**d**) Brassinolide content in plants grown under Fe-sufficient (white bars) and -deficient (black bars) growth conditions. Data shown are means ± standard error (n = 3). Different letters indicate significant differences (*p* ≤ 0.05) among treatments determined with two-way ANOVA followed by a Tukey’s test.

Although DLI-ESI-MS provides the possible composition of the compounds with minimal sample preparation, the exact metabolite identification is limited by the lack of fragmentation data or device accuracy (~0.3 Da) [6]. Therefore, mass spectrometry coupled with a separation technique such as GC, can provide identification with a high level of confidence based on the comparison of the retention time with the appropriate standard, and in addition, it allows calculation of the concentration of the compounds in the sample. In our study, we observed variations in the intensity of many ions in the mass spectra obtained from plants treated with UMCV2. Three of the *m*/*z* ions were putatively identified as components of the BRs biosynthesis (Table 2); we observed that the signal 419.15 *m*/*z*, which is a direct precursor of brassinolide [37] mainly increased in plants inoculated with the UMCV2 strain. Thus, we decided to confirm by GC-SIM-MS whether the volatiles emitted by *A. agilis* UMCV2, stimulate the production of brassinolide in *M. truncatula*, as it is the most bioactive form of BRs in plants. For this, we acetylated the molecule to change the analyte properties, which improved the identification capability of brassinolide (Figure 6a–c).

BRs are endogenous plant hormones that are essential for plant growth and development. Additionally, BRs are involved in sensing and responding to mineral deficiency stress. A factorial analysis showed that Fe deficiency stress, as well as microbial volatile factors significantly increased the content of epibrassinolide in the plant (*p* = 0.0039 and *p* = 0.0036, respectively); and the interaction of both factors was also statistically significant (*p* = 0.0153). Plants grown in Fe-deficient conditions and inoculated with UMCV2 showed a ten-fold higher accumulation of brassinolide relative to controls (Figure 6d). This result suggests that *A. agilis* induce the synthesis of brassinolide in the plant as a part of the mechanism for Fe stress tolerance; thus, the quantification of this phytohormone in the plants may serve as a reference of the beneficial effects of rhizobacterium to plants.

To summarize the information obtained in this study, a hierarchical cluster analysis was constructed for each treatment (Figure 5a,b). The metabolic heat maps based on DLI-ESI-MS data visually displayed differences between samples in the intensity of the selected *m*/*z* ions. Remarkably, two sets of ions were identified that accumulated under conditions of Fe deficiency, one of them was composed of the following ions 299.18, 185.22, 351.23, 189.26, 222.07, and 269.06 *m*/*z*, and the other was set by 300.18, 314.26, and 371.07 *m*/*z* whereas the remaining ions on the heat map diminished (Figure 5a); therefore, these ions could be considered as biomarkers of the plants’ Fe nutritional status.

Conversely, differences were observed in the metabolite profiling of plants exposed to microbial volatiles. Uninoculated plants and those inoculated with the L264 strain shared the same conglomerate, verifying that the volatiles from the commensal bacterium did not have an effect on the overall profile of plant metabolites produced in response to inoculation. Ions obtained from plants inoculated with the UMCV2 strain formed a separate conglomerate, and ions 87.41, 88.08, and 88.33 *m*/*z* presented the strongest differences, suggesting an important role during the interaction between *M. truncatula* and *A. agilis* UMCV2 (Figure 5b). 

## 3. Discussion

Fe is an important metal in photosynthesis. Additionally, Fe is used ubiquitously in oxidation-reduction processes [13]. These important roles make Fe an essential micronutrient for plant fitness. As a consequence, Fe deficiency is a major constraint for agricultural quality and production, eventually affecting human health via the food chain [16]. Fe scarcity leads to the activation of sophisticated mechanisms to maintain cellular Fe homeostasis. Legumes are classified as Strategy I plants, which undergo biochemical changes to increase the capacity for Fe uptake via the roots and Fe solubility in the soil [11]. In this study, we used DLI-ESI-MS and putatively identified several compounds in plants that are commonly accepted to be associated with biochemical responses and adaptation strategies under Fe deficiency conditions. Furthermore, this analytical tool showed sensitivity in discriminating plants grown under Fe-sufficient or -deficient conditions, providing evidence that the plants used in the study were metabolically stressed due to a lack of Fe. For example, we found that the mass spectra from Fe-deficient plants presented decreased signals for protochlorophyllide a (613.36 *m*/*z*) compared with control plants. The lower concentration of chlorophylls caused leaf yellowing, which is an important visible symptom of Fe deficiency in plants. In addition, the signal for 1-18:3-2-18:3-monogalactosyldiacylglycerol (797.49 *m*/*z*) also decreased. Monogalactosyldiacylglycerol is a major lipid component of chloroplast membranes and acts directly in several important plastid roles, particularly during photosynthesis [26,27]. Fe deficiency decreased the signals of other lipids including, phospholipid dioleoylphosphatidylcholine (808.38 *m*/*z*), which is a component of cell membranes, and 9,10-epoxy-18-hydroxystearate (314.26 *m*/*z*), which is involved in cutin biosynthesis [46,47]. 

In addition, we found that six signals were increased due to Fe deficiency which, according to the heat map, could be used as biomarkers to distinguish between Fe treatments. Two of these are precursors for riboflavin biosynthesis, 1-deoxy-l-glycero-tetrulose 4-phosphate (185.22 *m*/*z*) and 5-amino-6-(d-ribitylamino) uracil (299.18 *m*/*z*), which are subsequently transformed into FAD. Accumulation of riboflavin was observed in *M. truncatula* plants grown under Fe-deficient conditions, with or without CaCO_3_ as a source of alkaline pH stress. The root protein profile showed the de novo accumulation of 6,7-dimethyl-8-ribityllumazine synthase (DMRLs) and GTP cyclohydrolase II (GTPcII); these proteins are involved in riboflavin biosynthesis, suggesting that the riboflavin biosynthetic pathway is upregulated under conditions of Fe deficiency [11]. Since flavin compounds are exported and accumulate in Fe-deficient roots, different roles have been proposed for riboflavin, including as an electron donor either for enzymatic Fe (III) reduction, as a cofactor, or as a metal chelator [8,11]. 

Increased signaling was also observed for compounds of different families such as polyamines, coumarins, and terpenes; all of which have different roles in the adaptation of plants to the environment and for overcoming stress conditions. One signal corresponded to norspermine (189.26 *m*/*z*), a polyamine previously identified in *Medicago* plants [48], which accumulated in response to Fe deficiency, inducing ferric-chelate reductase activity and the expression of genes related to Fe uptake [49]. The coumarin (+)-marmesin (269.06 *m*/*z*) has antioxidant properties [50], as well as the tetraterpenoid crocetin (351.23 *m*/*z*) [14], which may protect plants from damage induced by oxidative stress in response to Fe deficiency [9]. Finally, the signal for the putative ion identified as L-histidinol phosphate (222.07 *m*/*z*) also increased. This compound is a precursor of histidine; however, its role in plants under stress caused by Fe deficiency has not been fully explored. The chemical properties of the imidazole side group allow this amino acid to participate in acid-base catalysis, and in the co-ordination of metal ions [51].

PGPR application has become an increasingly common practice as part of an agricultural strategy to alleviate plant abiotic stresses in the field. The use of PGPR will help to address the challenges of producing food for a growing human population in a sustainable and environmentally friendly manner. From this perspective, we have studied the effects of volatiles from the rhizobacterium *A. agilis* UMCV2 on the growth and development of *M. truncatula* in Fe-sufficient and -deficient growth media [23]. In a previous study, we found that the UMCV2 strain induces iron acquisition mechanisms in this Strategy I plant, including rhizosphere acidification, ferric chelate reductase activity, and Fe content in plants. In the present study, using DLI-ESI-MS and the RF model, we found that volatiles from the UMCV2 strain favor the accumulation of flavonoids in leaves under conditions of Fe sufficiency and deficiency, particularly kaempferol (287.19 *m*/*z*). Legumes are a source of flavonoids and have a beneficial effect on human health [52]; however in plants, flavonoids have diverse roles; for example, flavonoids reduce Fe (III) to Fe (II), reduce the production of reactive oxygen species (ROS), quench ROS, have antifungal activity, chelate ions of transition metals, and quench cascades of free-radical reactions in lipid peroxidation. Besides, due to their low redox potential, they can also reduce potent free radicals (superoxides, alkyl radicals, hydroxyl radicals) [45]. Last, they are involved in plant-microbe interactions signaling, in particular, in symbiotic bacteria stimulating root colonization [10,42,43,44]. 

Besides kaempferol, five other signals, 87.41, 88.08, 88.33, 97.83, and 98.04 *m*/*z*, which correspond to 3-pentanone, pyruvate, 4-aminobutanal, glycolate, and *N*-monomethylethanolamine, respectively, were grouped in the same conglomerate in the heat map, indicating that these compounds are metabolite markers that are specifically produced in response to the presence of the UMCV2 strain, compared with uninoculated plants and those treated with the commensal rhizobacteria L264. These compounds have previously been reported in plants subjected to different kinds of abiotic stresses, acting as signaling molecules that regulate many cellular processes, such as plant growth/development and acclimation responses to stress [12,28,29,30,31,32,33]. Thus, these results suggest that a complex network of signaling events is activated during the interaction of *M. truncatula* with *A. agilis* UMCV2 via the emission of volatile compounds; this stimulates iron acquisition mechanisms and mediates cellular activity to alleviate plant stress. 

Finally, of the 30 most important ions shown in the RF model, we identified three compounds (5-dehydroepisterol, 6-deoxocathasterone, and 6-hydroxycastasterone) related to BRs synthesis [35,36,37,38]. These phytohormones regulate the growth and development of plants, and their involvement in the detection and response to Fe deficiency in plants has only recently emerged [15,17,53]. The exogenous application of BRs to stressed plants induces stress-tolerance mechanisms. Thus, it would be timely to conduct a detailed study to ascertain whether microbial volatiles can modulate BRs signaling pathways in plants grown under conditions of Fe sufficiency and deficiency, since we found that volatiles from *A. agilis* UMCV2 promote the growth and the synthesis of BRs in plants grown under Fe-sufficient and -deficient growth conditions. 

In summary, our findings show the usefulness of DLI-ESI-MS for studying the metabolic disturbances induced by Fe deficiency in plants; and also, it provided an integrated view of the cellular processes that occur following inoculation with PGPR and different metabolite markers could be identified as possible subjects for further studies. The combination of both mass spectrometry techniques allowed us to show that plants effectively sense the volatiles emitted by *A. agilis* UMCV2 and reconfigure their metabolic networks accordingly. It is probable that multiple mechanisms, including brassinosteroid production, are activated during plant-microbe interactions, either simultaneously or in succession to ameliorate plant stress. Currently, we are investigating the role of volatiles from *A. agilis* UMCV2 in the protection against oxidative stress and the production of certain flavonoids and BRs to mediate Fe stress responses. 

## 4. Materials and Methods

### 4.1. Biological Material and Growth Conditions

In this study, seeds of *M. truncatula* ecotype Jemalong A17 were scarified with 2 mL of concentrated sulfuric acid for 8 min and then rinsed with five washes of sterile deionized water to remove excess acid [54]. Later, seeds were superficially disinfected with 12% sodium hypochlorite for 2 min and rinsed five times with sterile deionized water. Seeds were placed on 0.6% agar plates (Phytotechnology, Shawnee Mission, KS, USA) with 0.6% sucrose and vernalized at 4 °C for 3 days. Germination was performed in a Percival growth chamber with a photoperiod of 16 h light/8 h dark, a luminous intensity of 6100 lx and a constant temperature of 22 °C.

After 3 days of germination, three seedlings were transferred to each 170 mL glass flask with 35 mL of Hoagland medium and 0.6% agar. The Hoagland base medium was supplemented with the following salts: 1020 ppm KNO_3_, 492 ppm Ca(NO_3_)_2_ × 4H_2_O, 230 ppm NH_4_H_2_(PO_4_), 490 ppm MgSO_4_ × 7H_2_O, 2.80 ppm H_3_BO_3_, 1.81 ppm MnCl_2_ × 2H_2_O, 0.08 ppm CuSO_4_ × 5H_2_O, 0.22 ppm ZnSO_4_ × 5H_2_O and 0.09 ppm Na_2_MoO_4_ × H_2_O. For the Fe-sufficient treatment, Hoagland medium was supplemented with 20 μM FeSO4, and for the Fe-deficient treatment, no source of Fe was added.

The UMCV2 strain (CECT-7743, Spanish Type Culture Collection, Valencia, Spain) was grown on nutrient agar (3 g L^−1^ of meat extract, 5 g L^−1^ peptone, and 1.5% bacteriological agar) at 22 °C. Also, we used the commensal rhizobacterium *Bacillus* sp. L264 as a control [55,56]. The L264 strain was maintained under similar conditions to UMCV2.

### 4.2. Plant-Bacteria Interaction through the Emission of Volatiles

A system with separate compartments was used (Figure 1a–f). Two days after transferring the plants to Hoagland media, 20 μL of each rhizobacteria (0.05 D.O._595nm_) was inoculated into a glass vial with 2 mL nutritive agar medium. For the treatment of the uninoculated plants, 20 μL of water was added instead of bacterial inoculum. Then, seedlings were allowed to grow in the growth chamber under the controlled conditions of light and temperature mentioned above. After 10 days, the seedlings were carefully removed from the medium, and the trifoliate leaves were harvested, immediately frozen with liquid nitrogen, and maintained at −80 °C. Other plants were dried to a constant weight at 68 °C for 7 days. Dry weight was analyzed using a factorial design, comprising of two factors (Fe availability with two levels and bacterial volatiles with three levels), followed by Tukey’s test (*p* ≤ 0.05).

### 4.3. Metabolite Extraction from M. truncatula Leaves 

Frozen leaves were lyophilized and 3 mg of dry tissue was ground in a Mixer Mill (MM 400-Retsch, Verder Scientific GmbH & Co. KG; Haan, Germany) at 30 Hz for 30 s. The extraction was carried out with 500 μL 75% methanol grade HPLC acidified with 0.5% formic acid, and samples were then sonicated for 30 min, centrifuged at 10,000 rpm for 10 min at 4 °C, and filtered with a 4 mm syringe filter sterile through a 0.22 μm pore size hydrophilic nylon membrane. The samples were directly injected into the DLI-ESI mass spectrometer without further pre-treatment.

### 4.4. Non-Targeted Metabolic Profiling by DLI-ESI Mass Spectrometry

The samples were analyzed on a SQ-Detector 2 spectrometer (ESI/APCI/ESCi, multimode, Waters, Milford, MA, USA) with the fabricant software MassLynx 4.1. The measurements were made with electrospray ionization in positive mode, a capillary voltage of 3 kV, a cone voltage of 30 V and an extractor voltage of 3 V, source temperature of 135 °C, desolvation temperature and flow of 250 °C and 250 L h^−1^, respectively, and cone gas flow of 50 L h^−1^. The RF lens was set to 2.5 V. In the analyzer section, a resolution LM and HM of 10 and 14.6, respectively, and an energy ion of −0.1 were used. The samples were injected with a flow rate of 10 μL min^−1^. The spectra were collected within the range 50–2000 *m*/*z*, the duration of the run was 1 min, and one scan was obtained per second. 

The spectra obtained were converted from the raw extension to mzXML using MSConverte 3.0 from the ProteoWizard Library open-source initiative (https://proteowizard.sourceforge.net). The software mMass version 5.5.0 [57] was used to subtract the noise from the spectra, normalize the base peak, and obtain an average mass spectrum. Using the R language (Version 3.4.1 https://www.rstudio.com/) and the MALDIquant package [58], a database was obtained in text format with the ions present in the mass spectra. The database was used for statistical analyses.

The chemical profiles of the leaves were compared using the hierarchical cluster analysis (HCA) approach and by generating heatmaps with the ion intensities. To determine the contribution of each ion, the Rattle packet [59] was used in the R interface to generate a Random Forest (RF) model for each variable (iron and bacterial volatiles). The RF algorithm consisted of training, validation, and test steps using *m*/*z* ions. To obtain the most important ions in the study, 500 decision trees were created using 70% of the samples; the remaining samples were used for validation (15%) and testing (15%). The importance of the ion variable was determined by measuring the mean decrease by the Gini index.

### 4.5. Identification of Significant Ions

The most important ions, according to the RF algorithm, were putatively identified using SpiderMass software [60] and a *M. truncatula* metabolite database (PlantCyc database, https://www.plantcyc.org/) with a tolerance of ± 0.35 *m*/*z*.

### 4.6. Brassinosteroids Determination

The presence of BRs in the samples was confirmed by GC-MS (Agilent, Foster City, CA, USA) analysis. Each treatment consisted in three composed samples of three plants. The extracts were evaporated to dryness under a stream of nitrogen in a reaction vial. Then, they were treated with acetic anhydride (1.5 mL) and dichloromethane (1 mL), and heated at 75 °C for 90 min. Acetylation decreases the boiling point of the phytohormone, improves the thermal stability in the GC injection port and allows a better chromatographic separation. After cooling, the acetylated sample was diluted with chloroform (2 mL) and washed with deionized water (4 mL) three times. The organic phase was recovered, dried over anhydrous Na_2_SO_4_, evaporated and re-dissolved in 50 µL chloroform for GC-selected ion monitoring-MS analysis (GC-SIM-MS). The molecular ion of the acetylated compound at 652 *m*/*z* was very weak and sometimes not observed. Thus, the fragmented ions used for the SIM-MS method were 480, 380, 350, 322, and 177 *m*/*z*. These ions have previously been reported as characteristic ions for the structural determination of brassinolide rings, which have been analyzed by electron impact MS detector [61]. In addition, the phytohormone was further confirmed by comparing the retention time in the extract to a pure epibrassinolide standard (SIGMA-ALDRICH, Saint Louis, MO, USA, catalog no. E1641). The standard was also acetylated and to estimate the amount of the compound in the sample, we constructed a calibration curve (R^2^ = 1).

The phytohormone (2 µL) was analyzed using an Agilent 6850 Series II gas chromatograph equipped with an Agilent MS detector (model 5973) (Agilent) and a 5% phenyl methyl silicone capillary column (HP-5 MS) (30 m × 0.25 mm I.D., 0.25 mm film thickness). The operating conditions were 1 mL min^−1^ of helium as the carrier gas, 300 °C as the detection temperature, and 270 °C as the injection temperature. The column was held for 3 min at 180 °C and programmed at 5 °C min^−1^ to reach a final temperature of 300 °C for 12 min. The ions were monitored after electron impact ionization (70 eV).

Brassinolide concentration was analyzed using a factorial design, which comprised of two factors (Fe-rich and -deficient media, and the presence and absence of volatiles from *A. agilis* UMCV2), followed by Tukey’s test (*p* ≤ 0.05).

## Figures and Tables

**Table 1:** The most important compounds detected in *Medicago truncatula* seedlings by DLI-ESI-MS and putatively identified by the SpiderMass software, which were able to differentiate between samples grown under Fe-sufficient and -deficient growth conditions.

*m*/*z*	Monoisotopic Mass (Da)	Ionization Mode	Compound Name	Function
189.26	188.20	[M + H]^+^	Norspermine	Stress
185.22	184.01	[M + H]^+^	1-Deoxy-l-glycero-tetrulose 4-phosphate	Riboflavin biosynthesis
222.07	199.03	[M + Na]^+^	l-Histidinol-phosphate	Histidine biosynthesis
269.06	246.09	[M + Na]^+^	(+)-Marmesin	Stress
300.18	299.15	[M + H]^+^	(*S*)-*N*-methylcoclaurine	Stress
613.36	612.22	[M + H]^+^	Protochlorophyllide a	Chlorophyll biosynthesis
299.18	276.11	[M + Na]^+^	5-Amino-6-(d-ribitylamino) uracil	Riboflavin biosynthesis
859.98	837.16	[M + Na]^+^	Butanoyl-CoA	Fatty acid beta oxidation
797.49	774.53	[M + Na]^+^	1-18:3-2-18:3-Monogalactosyldiacylglycerol	Chloroplast membrane lipid
314.26	313.24	[M + H]^+^	9,10-Epoxy-18-hydroxystearate	Cutin biosynthesis
371.07	348.12	[M + Na]^+^	Chelerythrine	Stress
808.38	785.16	[M + Na]^+^	Dioleoylphosphatidylcholine	Membranes lipid
351.23	328.17	[M + Na]^+^	Crocetin	Stress

**Table 2:** The most important compounds detected by DLI-ESI-MS in *Medicago truncatula* seedlings and putatively identified by the SpiderMass software that could differentiate between samples exposed to volatiles from L264, UMCV2 strains, and control.

*m*/*z*	Monoisotopic Mass (Da)	Ionization Mode	Compound Name	Function
88.08	87.01	[M + H]^+^	Pyruvate	Energy
266.20	265.11	[M + H]^+^	Thiamine	Stress
383.14	360.14	[M + Na]^+^	7-Deoxyloganate	Stress
933.53	932.53	[M + H]^+^	Notoginsenoside R1	Stress
319.20	296.31	[M + Na]^+^	Phytol	Constituent of chlorophyll
365.17	342.12	[M + Na]^+^	Galactinol	Stress
98.34	75.07	[M + Na]^+^	*N*-Monomethylethanolamine	Choline biosynthesis
291.15	268.07	[M + Na]^+^	Formononetin	Stress
353.22	352.18	[M + H]^+^	16-Hydroxytabersonine	Indole alkaloid biosynthesis
397.20	396.34	[M + H]^+^	5-Dehydroepisterol	Brassinosteroid biosynthesis
287.19	286.05	[M + H]^+^	Kaempferol	Stress
97.83	75.01	[M + Na]^+^	Glycolate	Photorespiration
467.07	466.37	[M + H]^+^	6-Hydroxycastasterone	Brassinosteroid biosynthesis
321.11	320.09	[M + H]^+^	4-Coumaroylshikimate	Flavonoid and phenylpropanoid biosynthesis
790.06	767.12	[M + Na]^+^	Coenzyme A	Fatty acid beta oxidation
275.13	274.08	[M + H]^+^	Fustin	Stress
320.20	297.24	[M + Na]^+^	18-Hydroxyoleate	Cutin, suberin and wax biosynthesis
88.33	87.07	[M + H]^+^	4-Aminobutanal	Stress
87.41	86.07	[M + H]^+^	3-Pentanone	Stress
419.15	418.38	[M + H]^+^	6-Deoxocathasterone	Brassinosteroid biosynthesis
